# Orthotopic Liver Transplantation of a SARS-CoV-2 Negative Recipient from a Positive Donor: The Border between Uncertainty and Necessity in a Pandemic Era- Case Report and Overview of the Literature

**DOI:** 10.3390/medicina59050836

**Published:** 2023-04-26

**Authors:** Gabriela Droc, Cristina Martac, Cristina Georgiana Buzatu, Miruna Jipa, Maria Daniela Punga, Sebastian Isac

**Affiliations:** 1 Department of Anesthesiology and Intensive Care I, ‘Fundeni’ Clinical Institute, 022328 Bucharest, Romania; gabriela.droc@umfcd.ro (G.D.); christtina_martac@yahoo.com (C.M.); cristina.buzatu944@gmail.com (C.G.B.); mirunaa.jipa@gmail.com (M.J.); pungadaniela@yahoo.com (M.D.P.); 2 Department of Anesthesiology and Intensive Care I, Carol Davila University of Medicine and Pharmacy, 020021 Bucharest, Romania; 3 Department of Physiology, Faculty of Medicine, Carol Davila University of Medicine and Pharmacy, 020021 Bucharest, Romania

**Keywords:** liver transplantation, SARS-CoV-2 positive donor, liver surgery, immunosuppression therapy

## Abstract

(1) *Introduction*: Liver transplantation represents the gold-standard therapy in eligible patients with acute liver failure or end-stage liver disease. The COVID-19 pandemic dramatically affected the transplantation landscape by reducing patients’ addressability to specialized healthcare facilities. Since evidence-based acceptance guidelines for non-lung solid organ transplantation from SARS-CoV-2 positive donors are lacking, and the risk of bloodstream-related transmission of the disease is debatable, liver transplantation from SARS-CoV-2 positive donors could be lifesaving, even if long-term interactions are unpredictable. The aim of this case report is to highlight the relevance of performing liver transplantation from SARS-CoV-2 positive donors to negative recipients by emphasizing the perioperative care and short-term outcome. (2) *Case presentation*: A 20-year-old female patient underwent orthotropic liver transplantation for Child-Pugh C liver cirrhosis secondary to overlap syndrome, from a SARS-CoV-2 positive brain death donor. The patient was not infected nor vaccinated against SARS-CoV-2, and the titer of neutralizing antibodies against the spike protein was negative. The liver transplantation was performed with no significant complications. As immunosuppression therapy, the patient received 20 mg basiliximab (Novartis Farmacéutica S.A., Barcelona, Spain) and 500 mg methylprednisolone (Pfizer Manufacturing Belgium N.V, Puurs, Belgium) intraoperatively. Considering the risk of non-aerogene-related SARS-CoV-2 reactivation syndrome, the patient received remdesivir 200 mg (Gilead Sciences Ireland UC, Carrigtohill County Cork, Ireland) in the neo-hepatic stage, which was continued with 100 mg/day for 5 days. The postoperative immunosuppression therapy consisted of tacrolimus (Astellas Ireland Co., Ltd., Killorglin, County Kerry, Ireland) and mycophenolate mofetil (Roche România S.R.L, Bucharest, Romania) according to the local protocol. Despite the persistent negative PCR results for SARS-CoV-2 in the upper airway tract, the blood titer of neutralizing antibodies turned out positive on postoperative day 7. The patient had a favorable outcome, and she was discharged from the ICU facility seven days later. (3) *Conclusions*: We illustrated a case of liver transplantation of a SARS-CoV-2 negative recipient, whose donor was SARS-CoV-2 positive, performed in a tertiary, university-affiliated national center of liver surgery, with a good outcome, in order to raise the medical community awareness on the acceptance limits in the case of COVID-19 incompatibility for non-lung solid organs transplantation procedures.

## 1. Introduction

Liver transplantation represents, nowadays, the standard of care for patients with end-stage liver disease or acute liver failure. The main causes for developing liver cirrhosis are alcohol abuse, chronic viral hepatitis, autoimmune hepatitis, primary biliary cholangitis, cryptogenic hepatitis, overlap syndrome, or Wilson disease [1]. The leading cause of liver cirrhosis depends on country-related socio-economic factors; in developing countries, the main cause is chronic hepatitis, while alcohol abuse represents the main cause in industrialized countries [1].

Due to the COVID-19 pandemic, the addressability of patients with chronic conditions, including various liver pathologies, to healthcare providers decreased while their conditions worsened [2]. The severe acute respiratory syndrome coronavirus type 2 (SARS-CoV-2) has caused millions of victims worldwide since its outbreak in 2020 not only from the virus itself but also from the lack of appropriate treatment for their chronic diseases [3,4]. Thus, in the context of a preexisting worldwide donor crisis, and despite various national strategies, the pandemic affected, even more, the organ donation process, in the absence of acceptance patterns [5].

As the COVID-19 pandemic continued, major abdominal surgeries, including liver transplantation suffered a delay due to the need to find appropriate SARS-CoV-2 negative donors [6]. Data from the literature points out that cirrhotic patients or patients with advanced liver disease are prone to complications and death in this pandemic context [7]. Moreover, COVID-19 acts as a systemic disease, as it affects the lungs, kidneys, heart, brain, and liver [8,9]. The gastrointestinal tract and liver represent also important features of the disease [8]. Due to its prolonged shedding from the gastrointestinal tract, as stool samples from symptomatic and even asymptomatic patients have shown, the virus could reach through portal circulation in the liver [10,11]. Hepatic cell injury could result from either a direct viral infection, the antiviral drugs cytotoxicity, or the inflammatory response of the liver immune system [12].

Consequently, liver transplant recipients could have an even higher morbidity risk because of their fragile immune state and particular liver-specific tropism of the virus. The data from the literature revealed, however, conflicting results [13,14]. We have also previously shown that patients infected with SARS-CoV-2 following the liver transplantation surgery had good outcomes, and the survival rate was the same as for those without COVID-19 [15].

The immunosuppression therapy, following liver transplantation, involves a combination of drugs like corticosteroids, calcineurin inhibitors (CNI) (cyclosporine or tacrolimus), and antiproliferative agents (mycophenolate mofetil—MMF) according to local guidelines [16]. Tacrolimus could offer protection and lower mortality in SARS-CoV-2-positive liver recipients [17,18].

Even if the lungs represent the main transmission gateway of the SARS-CoV-2 virus, the uncertainty of infection through non-lung solid organ transplantation procedures remains [19]. The persistence of viral particles in the blood and endothelium could influence the decision to exclude SARS-CoV-2-positive donors from the non-lung solid organ transplantation [20].

Since evidence-based acceptance guidelines for non-lung solid organ transplantation from SARS-CoV-2-positive donors are lacking, and the risk of bloodstream-related transmission of the disease is debatable, some specialized surgery centers perform transplantation surgery using solid organs from SARS-CoV-2-positive donors [21,22,23,24].

This case report aims to highlight the relevance of performing liver transplantation from a SARS-CoV-2-positive donor to a negative recipient by presenting the perioperative care and outcome. Furthermore, this case should raise the clinician’s awareness in extending the pool of eligible liver donors in order to include those with present COVID-19 disease who check all the other mandatory requests.

## 2. Case Presentation

A 20-year-old female patient, 65 kg, 171 cm, underwent orthotopic liver transplantation for Child-Pugh C liver cirrhosis secondary to overlap syndrome from a SARS-CoV-2-positive donor.

The donor was a 16-year-old female patient, a victim of a car accident that, due to severe traumatic brain damage, was declared brain dead 48 h after admission. No other chronic condition was observed in her medical records. The vaccination status against SARS-CoV-2 was unknown. Furthermore, the donor did not show any pulmonary complications during the ICU stay, despite the positive PCR test for SARS-CoV-2 from the upper airway tract at admission. Since all criteria for organ harvesting were met, the medical team proceeded without any additional blood sampling in accordance with the national guidelines for solid organ transplantation.

The recipient’s preoperative model for end-stage liver disease (MELD) score was 17 points. The anamnesis revealed that the patient was not vaccinated for SARS-CoV-2, nor did she get the disease. The titer of SARS-CoV-2 neutralizing antibodies against the spike protein was undetectable before transplantation, as was the PCR test from the upper airway tract. Her medical records revealed an episode of upper digestive hemorrhage due to variceal rupture one year before surgery.

**Figure 1:**
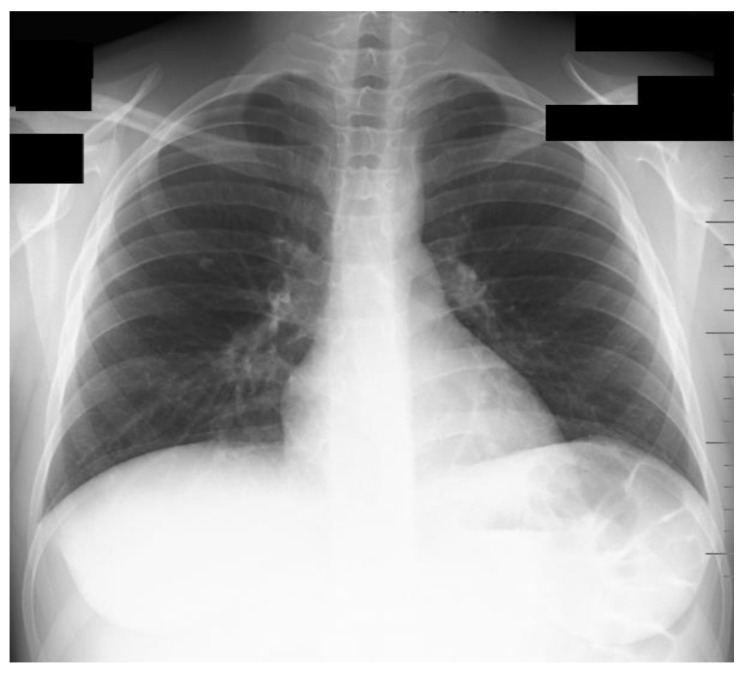
Chest X-ray before surgery (anteroposterior view).

The preoperative blood sample analysis revealed cirrhosis-related pancytopenia (mild leucopenia, moderate normochromic and normocytic anemia, moderate thrombocytopenia), cirrhosis-specific coagulopathy (International Normalized Ratio of 1.95, an activated partial thromboplastin time of 62.7 s, normal range 23–36 s, prothrombin time of 25 s, normal range 10.4–14.3 s, and fibrinogen levels of 154 mg/de, normal range 200–400 mg/dL). Biochemical results revealed elevated aspartate aminotransferase (160 U/L, normal range 0–34 U/L) and cholestasis (total bilirubin of 2 mg/dL, normal range 0.1–1.2 mg/dL, alkaline phosphatase of 323 U/L, normal range 43–132 U/L and gamma-glutamic transferase of 81 U/L, normal range 0–38 U/L). The preoperative chest X-ray revealed no structural changes (Figure 1).

Since the donor did not manifest any gastrointestinal symptoms related to a possible SARS-CoV-2 infection, no targeted liver biopsy was performed.

The patient underwent standard intravenous induction using fentanyl (Chiesi Pharmaceuticals GmbH, Wien, Austria), propofol (Fresenius Kabi GmbH, Graz, Austria), and succinylcholine (Takeda Austria GmbH) in accordance with the local guidelines. General anesthesia was maintained with sevoflurane (Abbvie Deutschland GmbH & Co., Ludwigshafen, Germany), fentanyl, and rocuronium (N.V. Organon, Oss, Holland). The respiratory and hemodynamically parameters were monitored continuously during the procedure [25]. The urine output was recorded hourly while the hemostasis and metabolic changes were monitored and corrected intermittently, at the discretion of the clinician, using thromboelastometry and blood–gas analysis.

Overall, the total fluid output consisted of 8000 mL ascites, 6000 mL blood loss, and 3100 mL urine, which was balanced with crystalloid infusion and albumin solution. The anhepatic phase lasted for 20 min. As primary prophylaxis against acute organ rejection syndrome, the patient received 20 mg basiliximab (Novartis Farmacéutica S.A., Barcelona, Spain) and 500 mg methylprednisolone (Pfizer Manufacturing Belgium N.V, Puurs, Belgium) intraoperatively. Considering the risk of lung-independent SARS-CoV-2 reactivation, the patient received antiviral therapy with remdesivir 200 mg/day (Gilead Sciences Ireland UC, Carrigtohill County Cork, Ireland) immediately after graft reperfusion (in the neo-hepatic stage).

Further, in the ICU, immunosuppression was maintained with tacrolimus (Astellas Ireland Co., Ltd. Killorglin, County Kerry, Ireland) and mycophenolate mofetil (Roche România S.R.L, Bucharest, Romania) at doses guided by daily tacrolinemia and blood sample analysis. A second dose of basiliximab (Novartis Farmacéutica S.A., Barcelona, Spain) was administered on the fourth postoperative day, in accordance with the local protocol. Further, the antiviral therapy with remdesivir 100 mg daily was maintained for 5 days.

**Figure 2:**
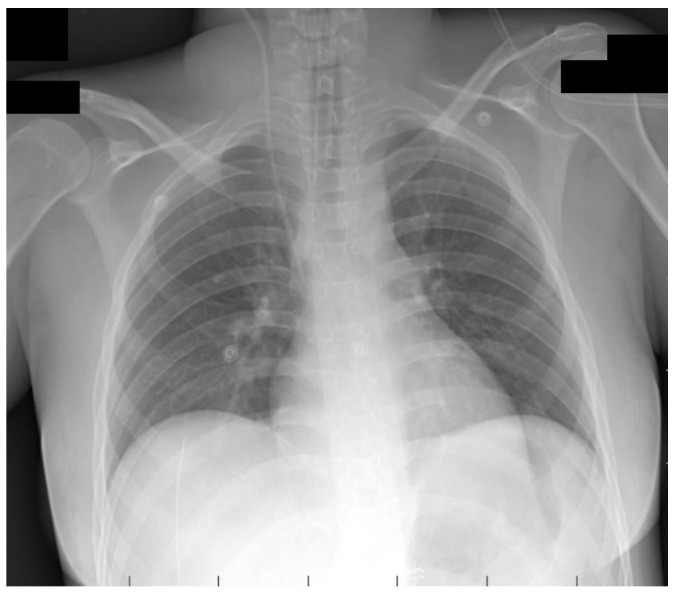
Chest X-ray seven days after surgery (anteroposterior view).

On the first postoperative day, the patient was weaned from the ventilator and repeated SARS-CoV-2 PCR test from the upper airway tract turned out negative. Additionally, on postoperative day 7, the titer of neutralizing antibodies against the spike protein was 1442 U/mL. The chest X-ray showed no structural changes (Figure 2).

As a differential diagnosis of the presence of postoperative neutralizing antibodies against the spike protein, we considered the passive immunity, once the new vascular liver anastomosis was made, or a non-lung-related SARS-CoV-2 reactivation. Since no other symptoms occurred, additional SARS-CoV-2-specific immunologic testing or liver biopsy were not needed.

The patient had a favorable postoperative outcome, without any clinical or biological signs of SARS-CoV-2 infection. She was discharged from the ICU facility seven days later.

## 3. Discussion

The decision to recover organs from donors with active COVID-19 should evaluate the risk of virus transmission, severe COVID-19 in an immunosuppressed patient, the recipient’s mortality risk, and long-term allograft outcome. These risks must be balanced against the life-saving benefit of a liver transplant in patients with end-stage liver disease since no other evidence-based recommendations state against their use. Our patient was a young patient with no other severe associated comorbidity, which had a good outcome after surgery and no short-term COVID-19-related complications.

Moreover, the patient was not vaccinated against SARS-CoV-2 prior to surgery, which raises an ethical issue regarding the importance of preoperative immunization against SARS-CoV-2 in this pandemic milieu. Sufficient data highlights the need for preoperative immunization since postoperative immunosuppression could exacerbate any infectious disease, including COVID-19 [26]. Kates et al. analyzed ethically two perspectives of SARS-CoV-2 vaccination for transplant candidates: the mandatory vaccination for recipients in the light of a potential increase in the number of SARS-CoV-2-positive donors and the optional vaccination [26]. Mandatory vaccination could constrain patients’ autonomy, while the optional vaccination programs should be enforced with valid strategies to increase the patient’s acceptance for vaccination, time-dependent on the organ availability. Since no further isolation strategies are used in most countries worldwide, immunization approaches should be prioritized and regulated.

The predominant mechanism of transmission is contact with droplets of respiratory secretions from an infectious individual- aerosol transmission. Angiotensin-converting enzyme 2 (ACE2-R) is the receptor for SARS-CoV-2, which is expressed not only in the lung but also in the liver [12]. Since the virus can be detected in specimens from other sites, other transmission mechanisms must be considered. According to Jayalakshmi Vallamkondu et al., the entry of SARS-CoV-2 into host cells is mediated by the interaction between the spike proteins and Angiotensin-Converting Enzyme-2 (ACE-2) receptors causing endocytic entry of the virus [27]. ACE 2 receptor is strongly expressed in the liver (liver cells, bile duct cells, liver endothelial cells), but its presence alone does not predict organ infection.

According to H. Y. Lei et al., SARS-CoV-2 particles could be detected in liver tissue, using the RT-PCR technique [12]. Additionally, in vitro, experiments have shown the ability of SARS-CoV-2 to infect and replicate in liver tissue [28]. In addition, studies that used human liver ductal organoids have demonstrated that SARS-CoV-2 can also damage the liver tissue [29]. Furthermore, the endothelium is also a target cell for SARS-CoV-2 [20]. The virus can cause endothelial cell dysfunction, leading to increased permeability, and adherence to the blood vessel wall, thrombosis, and multiorgan injury. Our patient had no anti-spike antibodies before surgery, but tested positive 7 days after, with no other symptoms. Therefore, two options could explain a possible recipient infection: through either endothelial cells and blood preserved in the donor’s liver or directly through hepatocytes and cholangiocytes. Furthermore, passive immunity could also explain the immunological results.

The risk of blood-related transmission of SARS-CoV-2 is supported by the reports of the detection of viral RNA in the blood of some infected individuals [30]. In addition, no transfusion-acquired SARS-CoV-2 has been reported, even when the transfusion was made from infected donors [31]. Moreover, studies have shown that a minimum RNA load is required to establish the correlation between the presence of viral RNA in a biological sample and infectivity, which is rarely detected in the blood [30]. Therefore, very low levels of infectious SARS-CoV-2 particles affect other organs through blood. These studies conclude that the risk of transmission of SARS-CoV-2 through blood remains theoretical [31,32].

The outcome of SARS-CoV-2-infected patients after liver transplantation could depend also on the infection time point, considering that immunosuppression intensity is also time-dependent and related to the drugs used [15,33].

Immunosuppression represents usually a risk factor for severe COVID-19 in liver-transplanted patients. The antiproliferative agents (mycophenolate mofetil) decrease the clonal expansion of alloreactive T cells resulting in high viral load and increased mortality in experimental settings [34]. These data are, however, not confirmed in clinical settings [33,35].

Conversely, some drugs used for immunosuppression could be helpful in reducing COVID-19 severity. Tacrolimus seems to improve the survival rate for liver-transplanted patients with COVID-19 [36]. Consequently, its dose is kept the same after a liver transplant regardless of COVID-19 infection in patients under 70 years old [17]. Studies have shown that tacrolimus has an inhibitory effect on the viral replication of other coronaviruses [37]. The mechanism of action of calcineurin inhibitors could be the protein–protein interactions between SARS-CoV-2 and the human host proteins [38]. This could explain the reduced number of SARS-CoV-2-positive patients among liver transplant recipients and the less severe COVID-19 disease progression in solid organ transplant recipients compared to the immunocompetent population [18]. Even if mycophenolate mofetil should be resumed in cases of severe infections, our patient received intraoperatively a combination of corticosteroids and basiliximab, and for long-term immunosuppression, mycophenolate mofetil, and tacrolimus, in the absence of any other COVID-19-related symptoms and in accordance with our national guidelines for immunosuppression after liver transplantation.

Finally, SARS-CoV-2 infection could cause liver graft dysfunction. Immune-mediated cholangitis could be a common finding in long-COVID syndrome as well as in chronic graft failure after liver transplantation [39,40]. Those cases are difficult to distinguish, while the treatment is basically different: intensive immunosuppression and corticotherapy and liver transplantation, respectively. Our patient presented, however, a good short-term outcome, with no further immune-mediated complications, even if the anti-spike antibodies turned out positive postoperatively in the absence of any positive PCR COVID-19 test from the upper airway.

## 4. Conclusions

We revealed a case of a SARS-CoV-2 negative liver recipient, whose donor was SARS-CoV-2 positive, and whose surgery was performed in a tertiary, university-affiliated national center of liver transplantation and surgery, with a good outcome, in order to raise the medical community awareness on the border between uncertainty and necessity of COVID-19 incompatibility transplantation procedures. Moreover, vaccination strategies and screening for SARS-CoV-2 in liver transplant candidates should be further prioritized since the isolation of infected persons is no longer practiced in many countries. Finally, proactive graft recovery from SARS-CoV-2 positive donors could represent a valid option for select cases that could be beneficial for the recipient with proven immunity against it.

## Data Availability

Not applicable.
